# Glutamic acid promotes hair growth in mice

**DOI:** 10.1038/s41598-021-94816-y

**Published:** 2021-07-29

**Authors:** Carlos Poblete Jara, Beatriz de Andrade Berti, Natália Ferreira Mendes, Daiane Fátima Engel, Ariane Maria Zanesco, Gabriela Freitas Pereira de Souza, Renan de Medeiros Bezerra, Julia de Toledo Bagatin, Silvya Stuchi Maria-Engler, Joseane Morari, William H. Velander, Lício A. Velloso, Eliana Pereira Araújo

**Affiliations:** 1 Faculty of Nursing, University of Campinas, UNICAMP, Tessalia Vieira de Camargo St., 126, Campinas, SP 13083-887 Brazil; 2 Faculty of Medical Sciences, University of Campinas, UNICAMP, Campinas, Brazil; 3 Laboratory of Cell Signalling, Obesity and Comorbidities Research Center, University of Campinas, UNICAMP, Campinas, Brazil; 4 Department of Organic Chemistry, Institute of Chemistry, University of Campinas, UNICAMP, Campinas, Brazil; 5 University of Campinas, Campinas, Brazil; 6 School of Pharmaceutical Sciences, Clinical Chemistry and Toxicology Department, University of São Paulo, São Paulo, Brazil; 7 Department of Chemical and Biomolecular Engineering, University of Nebraska, Lincoln, NE USA

**Keywords:** Target identification, Drug development

## Abstract

Glutamic acid is the main excitatory neurotransmitter acting both in the brain and in peripheral tissues. Abnormal distribution of glutamic acid receptors occurs in skin hyperproliferative conditions such as psoriasis and skin regeneration; however, the biological function of glutamic acid in the skin remains unclear. Using ex vivo, in vivo and in silico approaches, we showed that exogenous glutamic acid promotes hair growth and keratinocyte proliferation. Topical application of glutamic acid decreased the expression of genes related to apoptosis in the skin, whereas glutamic acid increased cell viability and proliferation in human keratinocyte cultures. In addition, we identified the keratinocyte glutamic acid excitotoxic concentration, providing evidence for the existence of a novel skin signalling pathway mediated by a neurotransmitter that controls keratinocyte and hair follicle proliferation. Thus, glutamic acid emerges as a component of the peripheral nervous system that acts to control cell growth in the skin. These results raise the perspective of the pharmacological and nutritional use of glutamic acid to treat skin diseases.

## Introduction

Glutamic acid (GA) is the major excitatory neurotransmitter in the mammalian central nervous system^1^. GA receptors (Grin1, Grin2a, Gria2, and Grm1) and transporters (Slc1a1 and Slc1a2) have also been identified in the skin across different species, such as mice, rats, and humans^2–6^. Moreover, in histological analyses, glutamate has been identified in the epidermis, hair follicles and sebaceous glands^7^.

Several studies had shown that the skin performs as neuro-endocrine organ^8–10^ and its activities are mainly regulated by local cutaneous factors^9^. This interaction between skin and environment factors can regulate Central Nervous System (CNS) functions^11^. For instance, ultraviolet light absorption by the skin can upregulate neuroendocrine axes^10,11^ and it is suggested to modulate body weight^12–14^ and depression-like behaviour^15^. Specifically, UVB skin exposure stimulate corticotropin-releasing hormone protein production and gene expression in the hypothalamus^10^.

Previous reports have been identified both the glutamate receptors and specific glutamate transporters in epidermal keratinocytes^2^. Physiologically, glutamatergic signalling through N-methyl-D-aspartate (NMDA) receptor was previously shown to occur in hair follicle cells. GA signalling is essential for the innervation and differentiation of Grin1 positive Schwann cells during piloneural collar development in hair follicles^4^. Specifically, NMDA receptors are highly expressed in type I and type II terminal Schwann cells. These cells are circumferentially localized in the bulge border and cover most outer root sheath keratinocytes in the isthmus^4^.

In cell culture studies, NMDA induced an increase in the number of keratinocytes and in the intracellular calcium concentration^16^; whereas, in vivo studies have shown that the topical application of GA to wounded skin in diabetic rats increases the rate of wound closure by inducing collagen synthesis and crosslinking^17^. In addition, 1% L-glutamic acid-loaded hydrogels accelerated vascularization and macrophage recruitment in diabetic wound^17^. D-glutamic acid has also been shown to act on damaged skin by accelerating the barrier recovery^3^, altogether suggesting a positive effect in skin repair.

Because of the preliminary data suggesting that GA could act in the skin, we performed a search in patent databases and found five patent requests for the use of topical GA and derived molecules for hair growth stimulation (patent numbers: CN106580722A, KR20150110149A, USOO58O1150A, FR2939038B1, and PI9302024A). However, there are no studies reporting on the mechanisms mediating the actions of GA to stimulate hair growth or epidermal cell proliferation. Here, we hypothesized that GA could induce proliferation and promote skin cell viability. Using in vivo, ex vivo and in silico models, we show that GA promotes keratinocyte proliferation and hair follicle growth by mechanisms that involve the control of vascularization and apoptosis.

## Results

### Glutamic acid increases human keratinocyte viability and proliferation

**Figure 1:**
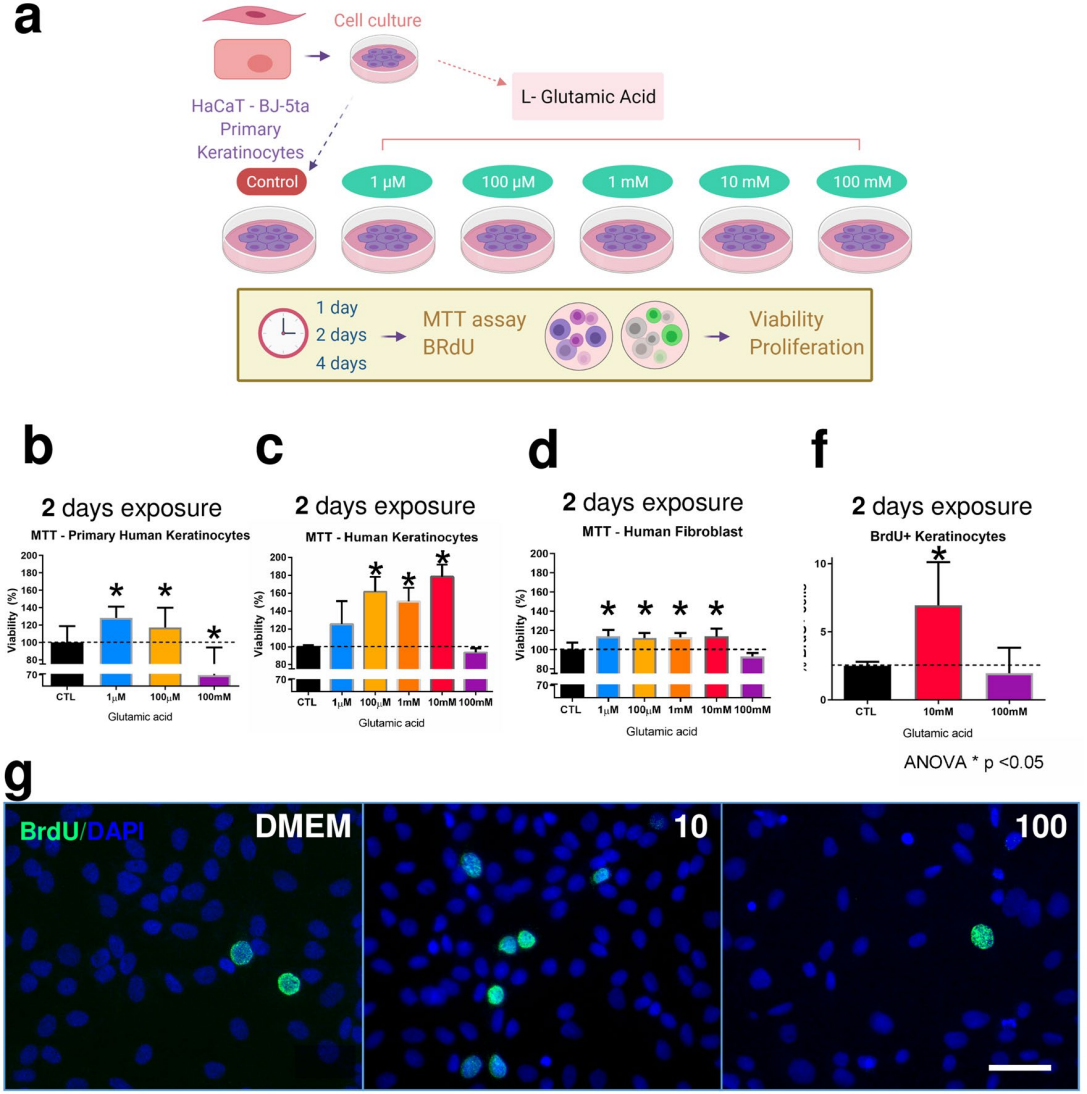
Effects of GA treatment in cell culture and in vivo. Experimental design of cell culture experiment using HaCaT, Primary human keratinocytes, and human fibroblast treated with different concentration of GA (**a**). MTT viability test results of Primary human keratinocytes with 2 days of Glutamic acid exposure (**b**). MTT viability test results of HaCaT with 2 days of Glutamic acid exposure (**c**). MTT viability test results of huma fibroblast with 2 days of Glutamic acid exposure (**d**). MTT viability test of primary keratinocytes in triplicate in 2 different experiments. MTT viability test of HaCaT keratinocytes in quadruplicate in 4 different experiments. MTT viability test of human fibroblast in quadruplicate in 2–3 different experiments. Immunostaining results of HaCaT keratinocytes treated with Glutamic acid 10 mM or 100 mM in DMEM compared to Control group treated with DMEM (**g**). Immunostaining results of HaCaT keratinocytes in triplicate in 4 different experiments, GA for 48 h and, finally, 3 h with BrdU, scale bar 50 μm. The proportion of BrdU-immunoreactive increased after exposure to GA 10 mM (**f**). Data is presented as mean ± SEM. N = 4 per group. p = 0.03 t-test Control versus GA 10 mM; p = 0.03 one-way ANOVA.

Firstly, we tested the hypothesis that GA could stimulate proliferation and survival of HaCaT, primary keratinocytes, and fibroblast, even in confluent culture conditions (Fig. 1a). All traces of foetal calf serum were removed from the medium to mitigate the effect of the growth factors present in the bovine serum. After two days of GA exposure, and even under 100% confluence conditions, keratinocyte viability and proliferation were increased. We showed that keratinocytes undergo a Gaussian distribution pattern of viability after GA exposure (Fig. 1b,c). After one day of treatment, GA (100 μM and 10 mM) increased HaCaT-keratinocyte viability (Supplementary Fig. 1a). These differences were higher after two days of GA exposure: the 100 μM, 1 mM, and 10 mM GA concentrations increased HaCaT keratinocyte viability within two days of treatment (Fig. 1c). Moreover, 1 μM and 100 μM GA concentrations increased primary keratinocyte viability within two days of treatment (Fig. 1b). Also, human fibroblast viability increased after one day (Supplementary Fig. 1c.) two days (Fig. 1d) and four days (Supplementary Fig. 1d.) of GA exposure under 100% confluence conditions. Conversely, we identified an excitotoxic concentration for keratinocytes at 100 mM GA but not in fibroblast. Keratinocytes treated with 100 mM GA decreased cell viability after one (Supplementary Fig. 1a) and four days of treatment (Supplementary Fig. 1b). As the 10 mM and 100 mM GA concentrations showed opposite effects in the viability test (proliferative and excitotoxic, respectively), we evaluated whether keratinocyte proliferation could be affected after two days of GA exposure (Fig. 1g). Consistent with the viability results, BrdU positive keratinocytes were increased in the 10 mM group (Fig. 1f,g).

### Topical glutamic acid decreases apoptotic related genes

**Figure 2:**
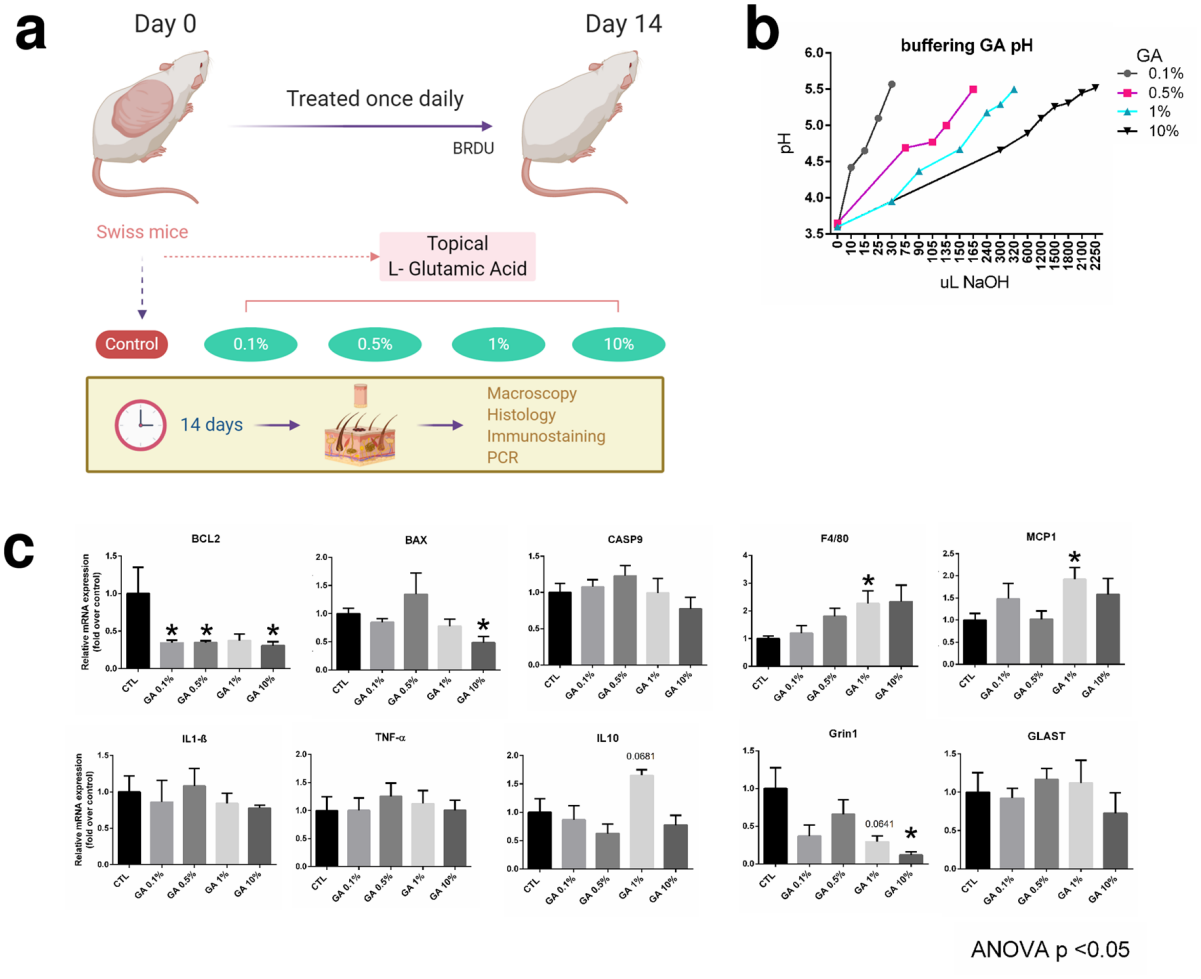
Topical glutamic acid in mice. Experimental design of Swiss mice treated topically one at day with different concentration of GA for 14 days (**a**). Different GA concentrations (Control, 0.1%, 0.5%, 1% and 10% GA) for topical animal treatment were equal to 5.5 pH (**b**). RT-PCR of Bcl2, Bax, Casp9, F4/80, Mcp1, Il1β, Tnfα, Il10, Grin1 and Glast genes from skin samples after 14 days of GA treatment (**c**). GAPDH was used as endogenous control. Data is presented as mean ± SEM * < p 0.05 ANOVA. 5–6 animals per group.

To determine whether the results obtained in cultured cells could be translated into an in vivo model, we employed four different concentrations of GA on the dorsal skin of Swiss mice (Fig. 2a). To understand how GA promotes proliferation and improves viability, we evaluated the expression of genes involved in apoptosis. There were reductions of Bcl2 gene expression in cells treated with GA 0.1%, 0.5% and 10% (Fig. 2c). BAX was decreased in cells treated with 10% GA (Fig. 2c). However, we found no differences in Casp9 expression (Fig. 2c). Next, we evaluated whether topical GA could stimulate the expression of genes related to inflammatory response. We found no differences in Il1-β, Tnf-α and Il10. However, F4/80, a macrophage marker, and Monocyte Chemoattractant Protein-1 (Mcp1) gene expression were increased after 14 days of 1% GA (Fig. 2c). Additionally, topical GA 10% decreased the expression of Glutamate Ionotropic Receptor NMDA Type Subunit 1 (Grin1) with no differences in Glutamate Aspartate Transporter 1 (Glast) expression (Fig. 2c).

### Topical glutamic acid accelerates hair growth in healthy mice

**Figure 3:**
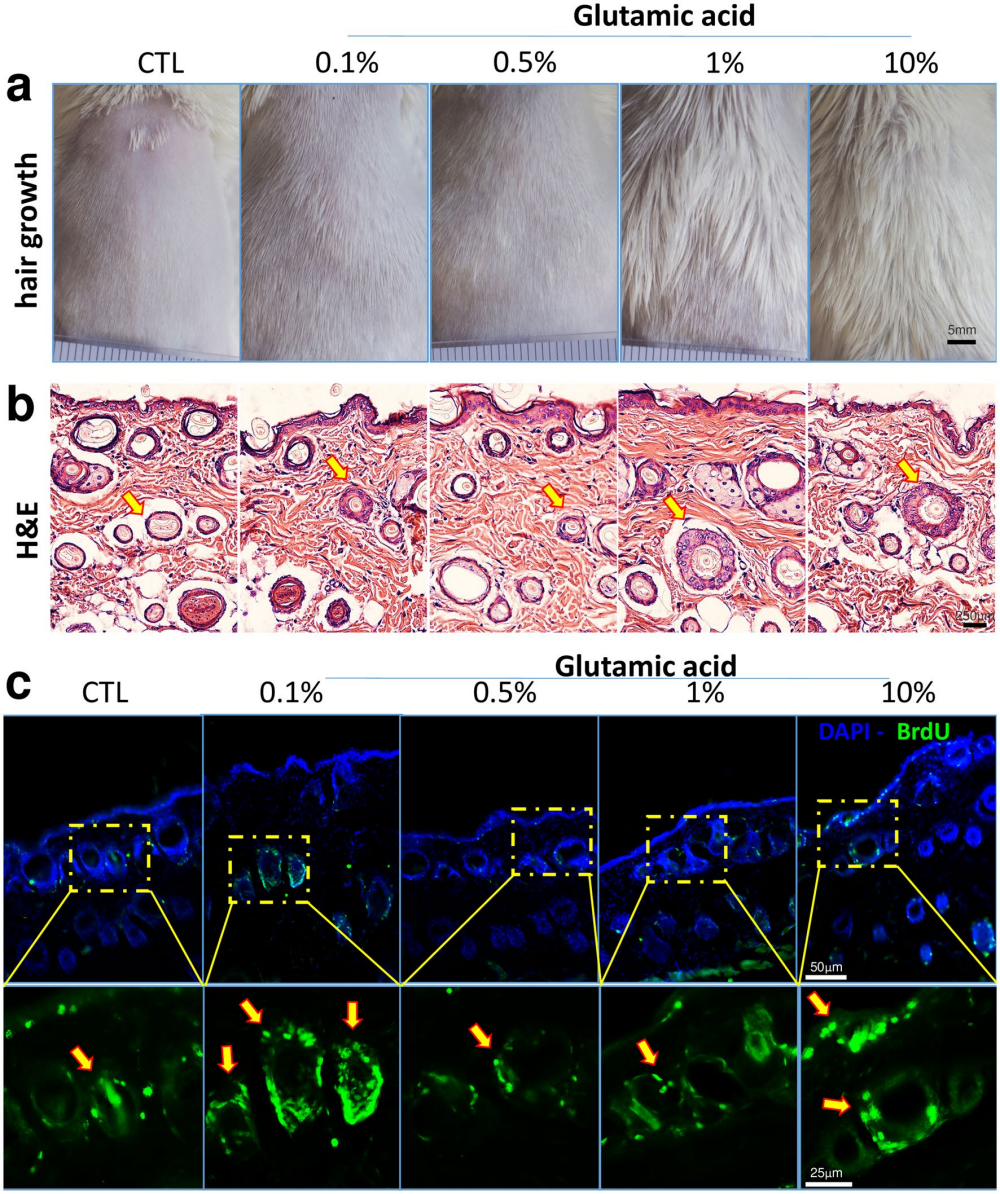
GA stimulates hair growth and increased BrdU + cells. Dose–response results of topical GA application on the dorsal region of mice with Vaseline (CTL) or 0.1%, 0.5%, 1% and 10% GA for 14 days (**a**–**c**). 5–6 animals per group. Hair growth effect after 14 days of GA treatment on the back of Swiss mice (**a**). Haematoxylin and Eosin (H&E) staining sample of the back of 14 day treated mice (hair follicle pointed with yellow arrows), scale bar 250 μm, samples from 3 different animals (**b**). Immunostaining results of skin samples treated 0.1%, 0.5%, 1%, and 10% Glutamic acid compared to Control group (Vaseline) (**c**). Immunostaining results of skin samples from 3 different animals, GA topical treatment for 14 days and, finally, 2 h with intraperitoneal BrdU. Yellow arrows indicate BrdU + cells.

**Figure 4:**
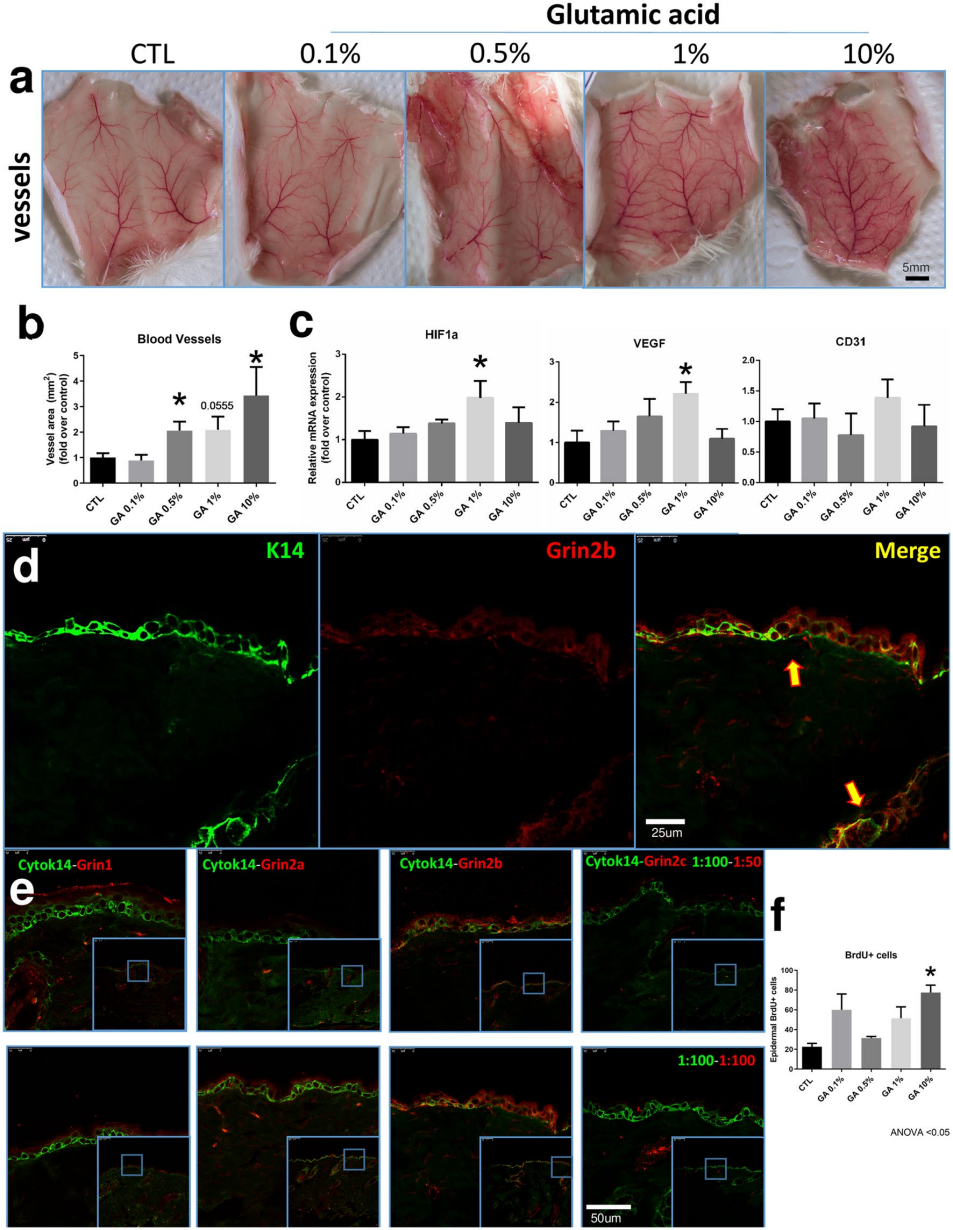
Topical GA and blood vessel. Skin samples treated for 14 days with topical GA topical. Upside-down back skin samples showing vessel differences between Vaseline (CTL) or 0.1%, 0.5%, 1% and 10% GA treatments (**a**). Quantification of blood vessel area after 14 days of vaseline (CTL) or 0.1%, 0.5%, 1% and 10% GA treatment (**b**). Gene expression of Hypoxia Inducible Factor 1 Subunit Alpha (Hif1a), Vascular Endothelial Growth Factor A (Vegf) and the Platelet and Endothelial Cell Adhesion Molecule 1 (Cd31) from full-thickness back skin after 14 days of GA treatment (**c**). GA receptor characterization in mice skin of different GA subunits (d-e). Immunostaining against NMDA Grin1, Grin2b, Grin2a and Grin2c GA receptor expressed in the epidermal layer of the skin of untreated mice (**d**–**e**). Yellow arrows indicate colocalization of K14 + Grin2b + cells (**d**). Quantification of positive BrdU epidermal and hair follicle cells of mice skin treated with vaseline (CTL) or 0.1%, 0.5%, 1% and 10% of GA (f). BrdU and quantitative PCR data are presented as mean ± SEM * < p 0.05 ANOVA. 5–6 animals per group.

Surprisingly, 1% and 10% GA accelerated hair growth after 14 days of topical treatment (Fig. 3a). Using photomicrographs, we also showed that GA increased external root sheath across all GA concentrations (Fig. 3b, Supplementary Fig. 1f.) with no hyperkeratosis effect. We also consistently identified increased BrdU positive cells in the hair follicles and epidermal layer after 14-days of GA topical treatment (Figs. 3c, 4f).

### Exogenous topical glutamic acid increased vascularization

We identified macroscopic differences in vascularization after 14 days of GA treatment. The 0.5% and 10% GA topical application increased skin vascularization (Fig. 4a,b). To further explore these findings, we evaluated whether GA could induce the expression of genes involved in vascular regulation. We found that 1% GA topical treatment increased Hypoxia Inducible Factor 1 Subunit Alpha (Hif1a), a master regulator of vascularization ^18,19^ (Fig. 4c). Also, 1% GA topical treatment increased the Vascular Endothelial Growth Factor A (Vegf), which induces proliferation and migration of vascular endothelial cells and is essential for physiological angiogenesis ^20–22^ (Fig. 4c). However, we found no difference in gene expression of CD31 after 14 days of topical GA treatment (Fig. 4c).

### Single cell RNA sequencing analysis showed differences in glutamate receptor and transporter localization between mice and human

**Figure 5:**
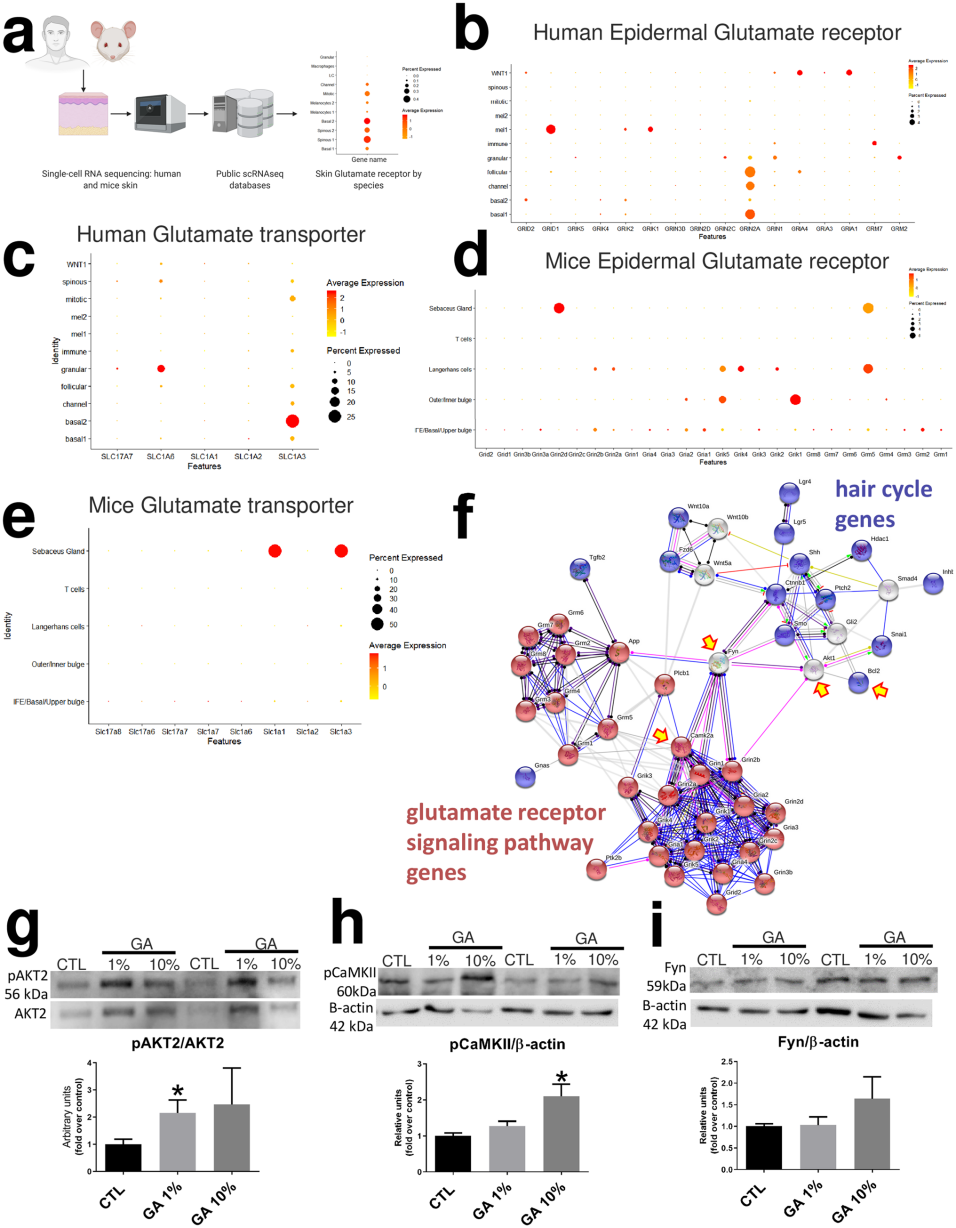
Cross-species skin GA receptor landscape using single-cell RNA sequencing. Generation of glutamic acid receptor landscape using public data reveals GA distribution at single cell resolution in mice and humans (**b**–**e**). Schematic representation of the single-cell RNA sequencing analysis using publicly available datasets from mice and human epidermal layers (**a**). Human Epidermal Glutamate receptors (**b**) and transporter expression (**c**). Mice Epidermal Glutamate receptors (**d**) and transporter expression (**e**). Glutamic acid receptor and hair cycle Protein–Protein Interaction Network performed with STRING V11 (**f**). Glutamic acid and hair cycle interactome were retrieved with the data-mining toolkit STRING. Closer interactors of glutamic acid and hair cycle ontologies (GO:0007215 and GO:0042633) were selected and categorized by coloured nodes. Yellow arrows indicate shared shell interactors, red nodes indicate glutamate receptor signaling pathway genes and blue nodes hair cycle genes (**f**). Display of cropped blots quantified to confirm the Protein–Protein Interaction Network prediction (**g**–**i**). Western blot analysis of AKT Phosphorylation (**g**), phospho-CaMKII (**h**) and Fyn quantification (**i**). Full-length blots are presented in Supplementary Fig. 3. Western blot data are presented as mean ± SEM * < p 0.05 ANOVA. 4–5 animals per group.

We evaluated glutamate receptor expression using immunostaining, quantitative PCR, and single-cell RNA sequencing techniques. We identified that NMDA receptor subunits Grin1, Grin2a, Grin2b and Grin2c are expressed in the skin (Fig. 4d,e), and Grin2b is expressed specifically in keratin 14 + cells (Fig. 4d,e). Due to the wide number of subunits (5 GA receptor families with 26 subunits), we used a single cell RNA sequencing approach to improve accuracy (Fig. 5a). Using public transcriptome libraries of skin tissue, we analysed ~ 73,000 mice and human epidermal cells from back (mice), foreskin, trunk, and scalp (human). This cross-species analysis showed a similar percentage (5%) of *glutamatergic epidermal population* in the skin (Fig. 5b,d). In humans, we identified NMDA receptors as the highest expressed subunits in the basal layer and hair follicular cell clusters, specifically the GRIN2A subunit (Fig. 5b). In addition, we identified melanocytes expressing Glutamate Ionotropic Receptor Delta Type Subunit 1 (GRD1) (Fig. 5b), granular cells expressing Excitatory Amino Acid Transporter 4 (SLC1A6) and basal layer cells expressing Excitatory Amino Acid Transporter 1 (SLC1A3) (Fig. 5c). In mice, we identified Grin2d (in the sebaceous gland) and Grik1 (in the hair follicle bulge) as the most expressed subunits (Fig. 5d). Additionally, we identified the expression of Excitatory Amino Acid Transporter 1 and 3 (Slc1a3 and Slc1a1) (Fig. 5e) in 50% of all sebaceous gland cells (Fig. 5e).

To predict a GA-mediated cell signalling pathway between GA pathway and hair follicle-related genes, we used computational interaction network analysis STRING^23^. In this way, we used *glutamate receptor pathway genes* and *hair cycle genes* ontologies (Supplementary Fig. 2). We found that GA receptors interact with hair cycle genes through the tyrosine-protein kinase *Fyn,* Ca 2 + /calmodulin-dependent protein kinase II *(CaMKII)* and protein kinase B (*Akt)* (Fig. 5f). Additionally, we found *Bcl2* as a common apoptotic regulator between both hair cycle and GA pathways (Fig. 5f). To confirm, we evaluated the protein expression of Fyn, CaMKII and Akt in the 14-day topical GA-treated mice (Fig. 5g–i)*.* We found no differences in Fyn quantification (Fig. 5i). However, we confirmed that AKT2 phosphorylation increased after 14 days of topical 1% GA treatment (Fig. 5g). Also, pCaMKII increased after 14 days of topical 10% GA treatment (Fig. 5h).

## Discussion

Currently, there are no studies describing GA treatment or even the effect of GA on hair growth or epidermal cell proliferation. However, upon searching through major patent agencies, we found five patents/patent requests claiming the benefits of topical GA (or derived molecules) for hair growth. One of these patents described the use of GA as a hair conditioner (patent number CN106580722A, China) for hair restoration and the prevention of alopecia. Another patent showed a Poly-Gamma-GA composition for preventing hair loss and promoting hair growth (KR20150110149A, Korea). In addition, there were synthetic compounds of GA attached to minoxidil for keratinocyte growth and hair growth in humans (USOO58O1150A, USA), a 2 to 12% GA topical cream for combating hair loss or alopecia in humans (FR2939038B1, France) and, finally, 42 different molecules derived from L-glutamic acid were described as hair growth promoters (PI9302024A, Brazil). However, reviewing all these patents/patents requests, we could find no description of the cellular mechanisms responsible for the stimulation of hair growth in response to GA application. Thus, our work provides experimental proof of a mechanistic link between GA and hair growth.

Here, we evaluated some of the potential mechanisms involved in effect of GA on hair growth. First, using a cell culture approach, we challenged 100% confluent primary human keratinocytes, HaCaT-keratinocytes, and human Fibroblast (in medium depleted of foetal bovine serum (FBS)/growth factor supply) to continue growing. Our results showed that GA increases the proliferation and viability of keratinocytes, even under these extreme conditions. Thus, GA could represent an interesting approach as a cell growth media supplement, replacing traditional supplements that are more expensive. This finding is further supported by data published previously, which shows that MK-801, an antagonist of the GA receptor (NMDA receptor), decreases the proliferation of primary human keratinocytes^2^ and also prevents hyperplasia induced by acetone^3^, suggesting an anti-proliferative effect.

The skin is a critical peripheral neuro-endocrine-immune structure that interact to central regulatory systems^24^. As a response, the skin can trigger cutaneous nerve endings to inform the CNS on changes in the epidermal or dermal environments to produce neural or immune responses at the local and systemic levels^24^. Human skin reacts to several neuropeptides and neurotransmitters by paracrine, autocrine, vasculature and nerves stimulus^25^. Primary and HaCaT keratinocytes are sources of Glutamic acid secreting ≈ 1 mM l-glutamic acid to the culture medium when 100% confluent^26^ suggesting a paracrine or autocrine stimulation. Indeed, major aspect of neuroendocine regulation in the skin, specifically the hypothalamic–pituitary–adrenal axis and melatoninoergic system in the skin was previously discussed^8^. GA has potent neurotoxic effects, and this could represent a challenge for either experimental or clinical use. Elevated amounts of GA lead to neuronal death in a process described as *excitotoxicity*^27–29^. GA transporters are a potent GA uptake system, acting as a neuronal compensatory response for excitotoxicity. GA transporters are known to prevent disproportionate activation of GA receptors by constantly removing GA from the extracellular space^30–32^. Here, we determined the excitotoxic GA concentration. In vitro, 100 mM GA decreased keratinocyte viability, and topical GA decreased Bcl2 and Bax expression. Altogether, our results support the excitotoxic effect of higher concentrations of pH-neutralized GA in keratinocytes.

To understand the exogenous GA effect on the skin, we explored the GA transporter landscape at single-cell resolution in human and mice skins (Fig. 5b,d). Additionally, we showed Slc1a3 expression using quantitative PCR, and a similar Slc1a3 (Glast) expression after exogenous GA application (Fig. 2c). Future research could help to identify the role of GA-induced excitotoxicity and the GA uptake system in the skin.

Regarding the GA receptors, different subpopulations of glutamatergic cells have been extensively described^33–36^. In the skin, previous reports identified the localization of GA receptors and transporters in the epidermis from rats and mice, as well as in human keratinocytes^2,3,5^. These studies showed similar cross-species characteristics: a smaller subpopulation of cells expressing receptors and transporters^2^. Consistent with these findings, here, we showed a small subpopulation of epidermal cells expressing GA receptors along the skin, with varying intensity (Fig. 5b,d). Our results suggest that these *glutamatergic keratinocytes* are responsive to exogenous GA stimulation.

Previous reports showed that vascularization increases during the anagen phase of the hair cycle and decreases during the catagen and telogen phases. This angiogenesis process was spatially correlated with the upregulation of VEGF^37^. Also, the hypoxia-inducible factor (HIF) has been shown to coordinate the up-regulation of multiple genes controlling neovascularization, such as Vegf^38^. Here, we showed that Hif1a and Vegf expression increased after 14 days of GA topical treatment on the back skin of mice with a remarkable change in angiogenesis, as previously shown^17^.

The Hypoxia-inducible factor-1α, encoded by the gene *Hif1a* showed to stimulate hair growth^39^ and some HIF-1α-stimulating agents significantly increase dermal papilla cell proliferation^40^. Minoxidil 2,4-diamino-6-piperidinopyrimidine3-oxide, a vasodilator used for the treatment of pattern hair loss, is a direct inhibitor of PHD-2 (prolyl-hydroxylase 2) which hydroxylates HIF-1α causing its degradation. Also, Minoxidil stimulates the transcription of hypoxia-response element genes such as VEGF^39–41^. Here, we showed different concentrations of GA with hair growth and angiogenic effects. Publicly available patents proclaim benefit by using high percentage of GA concentration for skin treatment and hair growth stimulation. The 10% GA treatment showed hair growth and angiogenesis stimulation, but no differences in Hif1a expression after 14 day of topical GA. Previous studies showed the ranges of GA saturation by specific GA concentration ^42–44^. In this way, we suggest that 10% GA topical treatment could achieve a post-acute signal saturation secondary to the time exposure and the Glutamic Acid concentration here presented. However, more studies are needed to elucidate a possible saturation and angiogenic effect of 14 days GA topical treatment on the back skin of mice.

A recent study supports that GA-mediated signalling could be involved in hair growth^45^. The authors showed that glutamine, a molecule similar to GA, controls the fate of stem cells in the hair follicle. The capacity of the outer root sheath cells to return to the stem cell state requires suppression of a metabolic switch from glutamine metabolism and is regulated by the mTORC2-Akt signalling axis^45^. Similarly, our result suggests that GA increases AKT phosphorylation and hair follicle cell stimulation. In this way, our results further suggest that GA activates the hair cycle by stimulating the stem cells to differentiate into the outer root sheath. However, future studies should describe the hair-follicle cycle modulation after topical GA treatments.

Taken together, the cell-based and experimental outcomes of this study provide a mechanistic advance in the characterization of GA-induced effects on hair growth and could become an attractive approach to treat hair growth disorders, or for aesthetic hair stimulation.

Further studies should focus on the relationship between skin disorders and GA and how GA, present in food, could impact on skin health.

## Methods

### Experimental animals

Eight-week-old male Swiss mice (n = 6) were obtained from the Breeding Animal Center of the University of Campinas. Animals were maintained under pathogen-free conditions in individual cages on a 12–12-h dark–light cycle, at 21–23 °C. Mice received food and water ad libitum. Mice were anesthetized with intraperitoneal injections (according to body weight), using ketamine hydrochloride 80 mg/kg and xylazine chlorhydrate 8 mg/kg. Hair was removed from the dorsal region (1.0 cm × 2.5 cm) of the anesthetized mice using a mechanical razor and depilatory cream (Veet). The dorsal region of all mice was carefully cleaned to remove any trace of Veet cream. Animal experiments were approved by The Animal Ethical Committee at the University of Campinas, Brazil (certificate of approval no. 4930–1/2018). All experiments were performed in accordance with the “Guide for the Care and Use of Laboratory Animals”, National Academy Press, 1996 guidelines of standard humane animal care. All the animal experiments have been performed following the ARRIVE guidelines.

### Topical glutamic acid treatment

The dorsal region of mice was treated once daily using different concentrations of GA. To ensure a uniform 200 μL treatment, we used different syringes preloaded with Vaseline (control), 0.1%, 0.5%, 1% or 10% GA (Supplementary Fig. 1c). The treatment was spread manually using gloves which were changing between each group. To avoid removal of the treatment, mice used Elizabethan collars 8 of 14 days of treatment.

### Topical glutamic acid composition

We made five different formulations: 0% (Control), 0.1% (6 mM), 0.5% (30 mM), 1% (60 mM) and 10% (600 mM) of GA. Table 1 shows the different composition of each treatment (Table 1). The pH of the formulations was adjusted using aqueous NaOH until the desired pH 5.5 was achieved (Fig. 1f). This pH value was chosen to resemble the skin surface pH^46^.

**Table 1:** Glutamic acid-based creams used in the in vivo experiments.

Compounds	Vaseline	6 mM (GA 0.1%)	30 mM (GA 0.5%)	60 mM (GA 1%)	600 mM (GA 10%)
L-Glutamic Acid	−	0.03 g	0.15 g	0.3 g	3 g
NaOH		35 µL	160 µL	320 µL	2.25 mL
Liquid Vaseline	2.77 mL	2.77 mL	2.77 mL	2.77 mL	2.77 mL
Solid Vaseline	24.78 g	24.75 g	24.63 g	24.48 g	21.75 g
Tween 20	200 µL	200 µL	200 µL	200 µL	200 µL
ddH_2_0	2.24 mL	2.215 mL	2.09 mL	1.91 mL	−
HCl	0.5 µL	−	−	−	−

Compound description of Control, GA 0.1%, 0.5%, 1%, and 10% w/w topical treatment.

### Primary keratinocytes isolation

Normal human skin from healthy donors were obtained by postectomy (University Hospital, University of São Paulo, São Paulo, Brazil). Keratinocytes were isolated and cultured as described previously^47,48^. Declaration of Helsinki Principles and approved by the Ethics Committees for Research (HU CEP Case No. 943/09 and CEP FCF/USP 534). Parents and/or guardians were informed and signed written consent.

### Cell culture MTT and BrdU

Human keratinocyte lineage (HaCaT) passage 27–30 were cultured in a 37 °C, 7% CO_2_ incubator with Dulbecco’s modified Eagle’s medium (DMEM) medium supplemented with 5% FBS to 100% confluence in 6-well plates. Primary keratinocytes passage 3–6 were cultured in a 37 °C, 7% CO_2_ incubator with KGM Gold Keratinocyte Growth Medium to 100% confluence in 6-well plates. Human Fibroblast cell line (BJ-5ta) passage 28–30 were cultured in a 37 °C, 5% CO_2_ incubator with 4:1 mixture of High glucose Dulbecco's Modified Eagle's Medium and Medium 199 supplemented with 10% FBS and 0.01 mg/ml hygromycin B, to 100% confluence in 6-well plates. We replaced the culture medium 2–3 times a week. A 3-4,5-dimethylthiazol-2-yl-2,5-diphenyltetrazolium bromide (MTT) assay was used to analyse cell viability, as previously described^49^. MTT solution was prepared in Krebs-HEPES buffer (10 mM HEPES, 1.2 mM MgCl_2_, 144 mM NaCl, 11 mM glucose, 2 mM CaCl_2_ and 5.9 mM KCl). After 100% confluence 6-wellplates, HaCaT and human fibroblast were incubated with the different concentrations of GA in DMEM without FBS for 1, 2 and 4 days. Primary keratinocytes were incubated with the different concentrations of GA in KGM Gold medium without growth factors for 2 days. After treatment, the medium was removed, MTT solution (0.5 mg/mL) was added to each well and the plates were incubated at 37 °C for 3 h. The solution was then removed and 300 μL of DMSO was added before being incubated in the dark with 60 rpm shaking. The absorbance was measured at a wavelength of 540 nm in a microplate reader (Globomax). HaCaT culture experiments were performed in quadruplicate in 4 different experiments. Primary keratinocytes culture experiments were performed in triplicate in 2 different experiments. Human fibroblast culture experiments were performed in triplicate in 2–3 different experiments. BrdU experiments were performed as previously described^26^. Briefly, to assess the effect of GA on cell proliferation, HaCaT human keratinocytes were maintained DMEM (Gibco) containing 4.5 g/L glucose, 4 mM L-glutamine, 100 units/mL of penicillin, 100 μg/mL of streptomycin and 10% FBS. Incubation conditions were 37 °C in 5% CO_2_/humidified air. HaCaT cells were plated on coverslips in 24-well plates (1 × 10^5^ cells/well) and exposed to GA for 48 h (10 and 100 mM) in DMEM without FBS. After treatment, cells were incubated with BrdU (10 µM, Sigma) for 3 h, then fixed with 4% PFA in 0.1 M PBS for 10 min at RT. For BrdU staining, cells were washed with PBS, and DNA was denatured with 1 N HCl for 1 h at RT. Cells were blocked for 1 h in blocking solution containing 10% goat serum and 0.2% Triton X-100 in PBS, followed by an incubation with primary (rat anti-BrdU; 1:200; Ab6326); and secondary (goat anti-rat FITC, 1:200; sc2011) antibodies prepared in 3% goat serum/0.2% Triton X-100 in PBS, and incubated overnight and for 2 h, respectively. The nuclei were labelled with DAPI, and coverslips were mounted onto glass slides for microscope imaging. Images were captured on fluorescence microscopy (Olympus BX41). The results of BrdU immunopositivity cells represent the average of 3 coverslips per experimental replicate, where 3 fields were imaged per coverslip and averaged. The number of immunopositive cells was quantified per image using the ImageJ software and are expressed as a percentage relative to the total DAPI nuclei.

### Animal photo documentation

Hair growth processes were photo documented using a D610 Nikon digital camera (Nikon Systems, Inc., Tokyo, Japan). We used a stand to secure a similar distance from the camera to the treated skin site, and the same person took the photos.

### Vessel analysis

Vascular density measurements were calculated from digital images obtained using a D610 Nikon digital camera (Nikon Systems, Inc., Tokyo, Japan). We used a stand to secure a similar distance from the camera to the upside-down back skin samples. Vascular density ratio was calculated vascular as follow: vessel area/total area * 100%^50^. The number of pixels were digitally determined by densitometry, using Image J software (National Institutes of Health).

### Histology

After 14 days of treatment, tissues were harvested and fixed by immersion in formaldehyde overnight. Any traces of formaldehyde were removed by 3 washes of PBS 1x. The tissues were processed in alcohol at different concentrations (70%, 80%, 95% and 100%), xylol and paraffin, before being fixed in paraffin blocks and sectioned at 5.0 μm. In total, 3 to 5 sessions were placed on microscope slides pre-treated with poly-L-lysine. To evaluate cell and extracellular matrix morphology, the skin sections were stained with haematoxylin and eosin (H&E). The sections were incubated with haematoxylin for 30 s, rinsed in water, incubated for 30 s with eosin, rinsed again in water, and dehydrated. The slides were mounted in Entellan® and then analysed; digital images were captured under bright-field microscopy.

### Protein–protein interaction networks

Protein functional interaction networks were performed using STRING v11. The default functional interaction network was configured to *evidence* meaning of network edges, *experiments,* and *databases* in active interaction sources. *Mus musculus* organism was visualized by known molecular action. We analysed two *Biological Processes* using the Gene Ontology Term from the Mouse Genome Informatics database: A permalink webpage of *Glutamate receptor* (GO:0,007,215) and *hair follicle* (GO:0,042,633). The gene ontologies interaction network is accessible through https://version-11-0.string-db.org/cgi/network.pl?taskId=lKUAbEZgGkVu for selected genes and https://version-11-0.string-db.org/cgi/network.pl?networkId=7i1fMIP01xqT for all genes.

### Single-cell RNA sequencing data acquisition, filtering, and processing.

In silico analyses were performed using a HP ENVY 17 Leap Motion SE NB PC notebook with 16 GB RAM and four-cores Intel i7 processor. Sample expression matrices (mice and humans) were downloaded from Gene Expression Omnibus and European Genome-phenome Archive: GSE67602 and EGAS00001002927. Cells were filtered by their total number of reads, by their number of detected genes and by their mitochondrial percentage. For mice, we used nFeature_RNA > 10 and < 6,000, nCount_RNA > 100 and < 50,000, percent.mt < 9.5 settings. For humans, we used nFeature_RNA > 100 and < 5,000, nCount_RNA > 100 and < 25,000 and percent.mt < 6 settings. Samples were processed in Seurat v3.1.5 using the default Seurat workflow. For clustering and visualization, we used the default Seurat pipeline gold standard and dot plot visualization. Cluster names were annotated to cell types according to original articles of Cheng et al. and Joost et al.^51,52^.

### Western blotting

For protein quantification, mice were treated topically 14 days with GA, once a day. Animals were anesthetized (ketamine hydrochloride 80 mg/kg and xylazine chlorhydrate 8 mg/kg) using *Labinsane*^53^, placed them in ventral decubitus position for cleaning the skin. Then, we harvested a 6 mm full-thickness skin sample^54^ from the back of each animal using a 6 mm biopsy punch. Immediately after the extraction, the tissues were stored in − 80 °C, until further analysis. The animals were sacrificed by anesthetic deepening. We used 5–6 animals per group. For the immunoblot experiments, the tissues were homogenized in Radioimmunoprecipitation assay buffer (150 mM NaCl, 50 mM Tris, 5 mM EDTA, 1% Triton X-100, 0.5% sodium deoxycholate, 0,1% sodium dodecyl sulfate, and supplemented with protease inhibitors). Insoluble materials were removed by centrifugation 11,000 rpm for 40 min at 4 °C, and the supernatant was used for protein quantification by the biuret reagent protein assay. Laemmli buffer (0.5 M Tris, 30% glycerol, 10% SDS, 0.6 M DTT, 0.012 bromophenol blue) was added to the samples. One hundred micrograms of proteins were separated by SDS-PAGE and transferred to nitrocellulose membranes (Bio-Rad) using a Trans-Blot SD Semi-Dry Transfer Cell (Bio-Rad) for 1 h at 17 V (constant) in buffer containing methanol and SDS. Blots were blocked in a 5% skimmed milk powder solution in TBST (1 × TBS and 0.1% Tween 20) for 2 h at RT, washed with TBST, and incubated with the primary antibodies for 24 h at 4 °C. The primary antibodies used were anti-pCaMKII (Abcam, ab32678) and anti-Fyn3 (Santa Cruz, sc-16). HRP-coupled secondary antibodies (1:5000, Thermo Scientific) were used for detection of the conjugate by chemiluminescence and visualization by exposure to an Image Quant LAS4000 (GE Healthcare, Life Sciences). Anti-β-actin (Abcam, ab8227) was used as a loading control. The intensities of the bands were digitally determined by densitometry, using Image J software (National Institutes of Health).

### Immunohistochemistry

Skin expressions of Grin1, Grin2a, Grin2b, and Grin2c were identified by immunohistochemical staining. Immunohistochemistry was performed using the skin samples (n = 5). Tissue samples were immersed in 4% formaldehyde, overnight. Tissue samples were washed three times with PBS 1x, cryopreserved in sucrose 20% for 3 days and 40% for 1 week. Samples were then embedded in OCT and sectioned using a cryostat (Leica CM1860). The sections (20 μm) were immunostained with the following primary antibodies: Grin1 (1:100, sc1467), Grin2a (1:100, sc1468), Grin2b (1:100, sc1469), Grin2c (1:100, sc9057), BrdU (1:200, ab6326) and keratin 14 (1:100, sc53253). VECTASHIELD with DAPI was used as a mounting medium for nuclear visualization. Images were obtained using a confocal microscope (Leica TCS SP5 II). For the in vivo BrdU experiment, we treated the mice intraperitoneally, as previously described^55^. Briefly, we applied one single injection of BrdU (150 mg/kg in buffer citrate) 3 h before skin harvest.

### Real-time quantitative polymerase chain reaction (RT-qPCR)

The total RNA content was extracted from the tissue using TRIzol reagent (Invitrogen). For each sample, two micrograms of RNA were reverse transcribed to cDNA, according to the manufacturer's instructions (High-Capacity cDNA Reverse Transcription Kit, Life Technologies). Gene expression analysis was performed via RT-qPCR using TaqMan Universal PCR Master Mix (7500 detection system, Applied Biosystems). The primers used were: Bcl2: Mm00477631_m1; Bax: Mm00432051_m1; Casp9: Mm00516563_m1; F4/80: Mm00802529_m1; Mcp1: Mm00441242_m1; Il1b: Mm00434228_m1; TNFa: Mm00443258_m1; Il10: Mm01288386_m1; Grin1: Mm00433790_m1; Glast: Mm00600697_m1; Hif1a: Mm00468869_m1; Vegf: Mm00437306_m1; and Cd31: Mm01242576_m1 (Thermofisher). Analyses were run using 4 μL (10 ng/μL) cDNA, 0.625 μL primer/probe solution, 1.625 μL H_2_O and 6.25 μL 2X TaqMan Universal MasterMix. GAPDH (Mm99999915_g1) was employed as a reference gene. Gene expression was obtained using the SDS System 7500 software (Applied Biosystems).

## Supplementary Information

Supplementary Information.

## References

[1] Zhou Y, Danbolt NC (2014). Glutamate as a neurotransmitter in the healthy brain. J. Neural Transm..

[2] Genever PG, Maxfield SJ, Kennovin GD, Maltman J, Bowgen CJ, Raxworthy MJ (1999). Evidence for a novel glutamate-mediated signaling pathway in keratinocytes. J. Invest. Dermatol..

[3] Fuziwara S, Inoue K, Denda M (2003). NMDA-type glutamate receptor is associated with cutaneous barrier homeostasis. J. Invest. Dermatol..

[4] Woo S-H, Baba Y, Franco AM, Lumpkin EA, Owens DM (2012). Excitatory glutamate is essential for development and maintenance of the piloneural mechanoreceptor. Development.

[5] Morhenn VB, Waleh NS, Mansbridge JN, Unson D, Zolotorev A, Cline P (1994). Evidence for an NMDA receptor subunit in human keratinocytes and rat cardiocytes. Eur. J. Pharmacol..

[6] Morhenn VB, Murakami M, O'Grady T, Nordberg J, Gallo RL (2004). Characterization of the expression and function of N-methyl-D-aspartate receptor in keratinocytes. Exp. Dermatol..

[7] Nordlind K, Johansson O, Lidén S, Hökfelt T (1993). Glutamate- and aspartate-like immunoreactivities in human normal and inflamed skin. Virchows. Archiv B..

[8] Zmijewski MA, Slominski AT (2011). Neuroendocrinology of the skin. Dermato-Endocrinol..

[9] Slominski A, Wortsman J (2000). Neuroendocrinology of the skin. Endocr. Rev..

[10] Skobowiat C, Slominski AT (2015). UVB activates hypothalamic–pituitary–adrenal axis in C57BL/6 mice. J. Investig. Dermatol..

[11] Slominski AT, Zmijewski MA, Plonka PM, Szaflarski JP, Paus R (2018). How UV light touches the brain and endocrine system through skin, and why. Endocrinology.

[12] Geldenhuys S, Hart PH, Endersby R, Jacoby P, Feelisch M, Weller RB (2014). Ultraviolet radiation suppresses obesity and symptoms of metabolic syndrome independently of vitamin D in mice fed a high-fat diet. Diabetes.

[13] Pulgar J, Waldisperg M, Galbán-Malagón C, Maturana D, Pulgar VM, Aldana M (2017). UV radiation impacts body weight, oxygen consumption, and shelter selection in the intertidal vertebrate Girella laevifrons. Sci. Total Environ..

[14] Londero JEL, Dos Santos CP, Segatto ALA, Passaglia SA (2017). Impacts of UVB radiation on food consumption of forest specialist tadpoles. Ecotoxicol. Environ. Saf..

[15] Han M, Ban J-J, Bae J-S, Shin C-Y, Lee DH, Chung JH (2017). UV irradiation to mouse skin decreases hippocampal neurogenesis and synaptic protein expression via HPA axis activation. Sci. Rep..

[16] Fischer M, Glanz D, William T, Klapperstuck T, Wohlrab J, Marsch W (2004). N-methyl-D-aspartate receptors influence the intracellular calcium concentration of keratinocytes. Exp. Dermatol..

[17] Thangavel P, Ramachandran B, Chakraborty S, Kannan R, Lonchin S, Muthuvijayan V (2017). Accelerated healing of diabetic wounds treated with l-glutamic acid loaded hydrogels through enhanced collagen deposition and angiogenesis: an in vivo study. Sci. Rep..

[18] Semenza GL (1998). Hypoxia-inducible factor 1: Master regulator of O2 homeostasis. Curr Opin Genet Dev..

[19] Chen L, Endler A, Shibasaki F (2009). Hypoxia and angiogenesis: Regulation of hypoxia-inducible factors via novel binding factors. Exp. Mol. Med..

[20] Ferrara N (2002). VEGF and the quest for tumour angiogenesis factors. Nat. Rev. Cancer.

[21] Rumney RMH, Lanham SA, Kanczler JM, Kao AP, Thiagarajan L, Dixon JE (2019). In vivo delivery of VEGF RNA and protein to increase osteogenesis and intraosseous angiogenesis. Sci. Rep..

[22] Leung DW, Cachianes G, Kuang WJ, Goeddel DV, Ferrara N (1989). Vascular endothelial growth factor is a secreted angiogenic mitogen. Science.

[23] Szklarczyk D, Gable AL, Lyon D, Junge A, Wyder S, Huerta-Cepas J (2019). STRING v11: Protein-protein association networks with increased coverage, supporting functional discovery in genome-wide experimental datasets. Nucleic Acids Res..

[24] Slominski AT, Zmijewski MA, Skobowiat C, Zbytek B, Slominski RM, Steketee JD. Sensing the environment: regulation of local and global homeostasis by the skin's neuroendocrine system. Adv. Anat. Embryol. Cell Biol. 2012;212:v, vii, 1–115. 10.1007/978-3-642-19683-6_1 PMC3422784 22894052

[25] Ramot Y, Böhm M, Paus R (2021). Translational neuroendocrinology of human skin: Concepts and perspectives. Trends Mol. Med..

[26] Fischer M, Glanz D, Urbatzka M, Brzoska T, Abels C (2009). Keratinocytes: a source of the transmitter L-glutamate in the epidermis. Exp. Dermatol..

[27] Dong X-x, Wang Y, Qin Z-h (2009). Molecular mechanisms of excitotoxicity and their relevance to pathogenesis of neurodegenerative diseases. Acta Pharmacol. Sin..

[28] Piña-Crespo JC, Sanz-Blasco S, Lipton SA. Concept of excitotoxicity via glutamate receptors. In: Kostrzewa RM, editor. Handbook of neurotoxicity. New York, NY: Springer New York; 2014. pp. 1015–38.

[29] Crabbé M, Dirkx N, Casteels C, Laere KV (2019). Excitotoxic neurodegeneration is associated with a focal decrease in metabotropic glutamate receptor type 5 availability: an in vivo PET imaging study. Sci. Rep..

[30] O’Donovan SM, Sullivan CR, McCullumsmith RE (2017). The role of glutamate transporters in the pathophysiology of neuropsychiatric disorders. NPJ Schizophr..

[31] Parsons MP, Vanni MP, Woodard CL, Kang R, Murphy TH, Raymond LA (2016). Real-time imaging of glutamate clearance reveals normal striatal uptake in Huntington disease mouse models. Nat. Commun..

[32] Paoletti P, Bellone C, Zhou Q (2013). NMDA receptor subunit diversity: impact on receptor properties, synaptic plasticity and disease. Nat. Rev. Neurosci..

[33] Tatti R, Bhaukaurally K, Gschwend O, Seal RP, Edwards RH, Rodriguez I (2014). A population of glomerular glutamatergic neurons controls sensory information transfer in the mouse olfactory bulb. Nat. Commun..

[34] Heikkinen AE, Möykkynen TP, Korpi ER (2009). Long-lasting Modulation of Glutamatergic Transmission in VTA Dopamine Neurons after a Single Dose of Benzodiazepine Agonists. Neuropsychopharmacology.

[35] Montardy Q, Zhou Z, Lei Z, Liu X, Zeng P, Chen C (2019). Characterization of glutamatergic VTA neural population responses to aversive and rewarding conditioning in freely-moving mice. Science Bulletin..

[36] Yamaguchi T, Qi J, Wang H-L, Zhang S, Morales M (2015). Glutamatergic and dopaminergic neurons in the mouse ventral tegmental area. Eur. J. Neurosci..

[37] Yano K, Brown LF, Detmar M (2001). Control of hair growth and follicle size by VEGF-mediated angiogenesis. J. Clin. Investig..

[38] Oladipupo S, Hu S, Kovalski J, Yao J, Santeford A, Sohn RE (2011). VEGF is essential for hypoxia-inducible factor-mediated neovascularization but dispensable for endothelial sprouting. Proc. Natl. Acad. Sci. USA.

[39] Houschyar KS, Borrelli MR, Tapking C, Popp D, Puladi B, Ooms M (2020). Molecular mechanisms of hair growth and regeneration: Current understanding and novel paradigms. Dermatology.

[40] Bukowiecki J, Pförringer D, Thor D, Duscher D, Brett E (2020). HIF-1α stimulators function equally to leading hair loss agents in enhancing dermal papilla growth. Skin Pharmacol. Physiol..

[41] Yum S, Jeong S, Kim D, Lee S, Kim W, Yoo JW (2017). Minoxidil Induction of VEGF Is Mediated by Inhibition of HIF-Prolyl Hydroxylase. Int. J. Mol. Sci..

[42] Holmes WR (1995). Modeling the effect of glutamate diffusion and uptake on NMDA and non-NMDA receptor saturation. Biophys J..

[43] Lee DW, Woo DC, Heo H, Kim KW, Kim JK, Lee DH (2020). Signal alterations of glutamate-weighted chemical exchange saturation transfer MRI in lysophosphatidylcholine-induced demyelination in the rat brain. Brain Res Bull..

[44] Lee DW, Woo CW, Woo DC, Kim JK, Kim KW, Lee DH (2020). Regional mapping of brain glutamate distributions using glutamate-weighted chemical exchange saturation transfer imaging. Diagnostics (Basel)..

[45] Kim CS, Ding X, Allmeroth K, Biggs LC, Kolenc OI, L’Hoest N, et al. Glutamine metabolism controls stem cell fate reversibility and long-term maintenance in the hair follicle. Cell Metabolism. 2020. 10.1016/j.cmet.2020.08.011 32905798

[46] Ali SM, Yosipovitch G (2013). Skin pH: From basic science to basic skin care. Acta Derm Venereol..

[47] do Nascimento Pedrosa T, Catarino CM, Pennacchi PC, de Moraes Barros SB, Maria-Engler SS,  (2021). Skin equivalent models: Protocols for in vitro reconstruction for dermal toxicity evaluation. Methods Mol. Biol..

[48] Hieda DS, Anastacio da Costa Carvalho L, Vaz de Mello B, Oliveira EA, Romano de Assis S, Wu J, et al. Air particulate matter induces skin barrier dysfunction and water transport alteration on a reconstructed human epidermis model. J Invest Dermatol. 2020;140(12):2343–52.e3. 10.1016/j.jid.2020.03.971 32339540

[49] Engel DF, de Oliveira J, Lieberknecht V, Rodrigues ALS, de Bem AF, Gabilan NH (2018). Duloxetine protects human neuroblastoma cells from oxidative stress-induced cell death through Akt/Nrf-2/HO-1 pathway. Neurochem. Res..

[50] Teplyi V, Grebchenko K (2019). Evaluation of the scars' vascularization using computer processing of the digital images. Skin Res Technol..

[51] Cheng JB, Sedgewick AJ, Finnegan AI, Harirchian P, Lee J, Kwon S (2018). Transcriptional programming of normal and inflamed human epidermis at single-cell resolution. Cell Rep..

[52] Joost S, Zeisel A, Jacob T, Sun X, La Manno G, Lönnerberg P (2016). Single-cell transcriptomics reveals that differentiation and spatial signatures shape epidermal and hair follicle heterogeneity. Cell Syst..

[53] Jara CP, Carraro RS, Zanesco A, Andrade B, Moreira K, Nogueira G, et al. A smartphone app for individual anesthetic calculation decreased anesthesia-related mortality in mice. bioRxiv. 2020:2020.09.09.289728. 10.3389/fvets.2021.651202 PMC8339260 34368269

[54] Wang X, Ge J, Tredget EE, Wu Y (2013). The mouse excisional wound splinting model, including applications for stem cell transplantation. Nat. Protoc..

[55] Aragona M, Dekoninck S, Rulands S, Lenglez S, Mascré G, Simons BD (2017). Defining stem cell dynamics and migration during wound healing in mouse skin epidermis. Nat. Commun..

