# Supplementary material for: Glutamic acid promotes hair growth in mice
Source: Sci Rep. 2021 Jul 29;11:15453. doi: 10.1038/s41598-021-94816-y (PMC8322389; doi:10.1038/s41598-021-94816-y)

SUPPLEMENTARY

GLUTAMIC ACID PROMOTES HAIR GROWTH IN MICE

Carlos Poblete Jara[1,3,5,7], Beatriz de Andrade Berti[1,3,5], Natália Ferreira Mendes[1,3,5], Daiane Fátima Engel [2,3,5], Ariane Maria Zanesco[2,3,5], Gabriela Freitas Souza[4,5], Renan de Medeiros Bezerra[1,3,5], Julia de Toledo Bagatin[6], Silvya Stuchi Maria-Engler[6], Joseane Morari[2,3,5], William H. Velander[7], Lício A. Velloso[2,3,5], Eliana Pereira de Araújo[1,3,5]

[1] Faculty of Nursing, [2]Faculty of Medical Sciences, [3] Laboratory of Cell Signalling, Obesity and Comorbidities Research Center, [4]Department of Organic Chemistry, Institute of Chemistry, [5]University of Campinas, Brazil, [6]School of Pharmaceutical Sciences, Clinical Chemistry and Toxicology Department, University of São Paulo, Brazil, [7]Department of Chemical and Biomolecular Engineering, University of Nebraska, Lincoln, NE, USA

Supplementary Figure 1

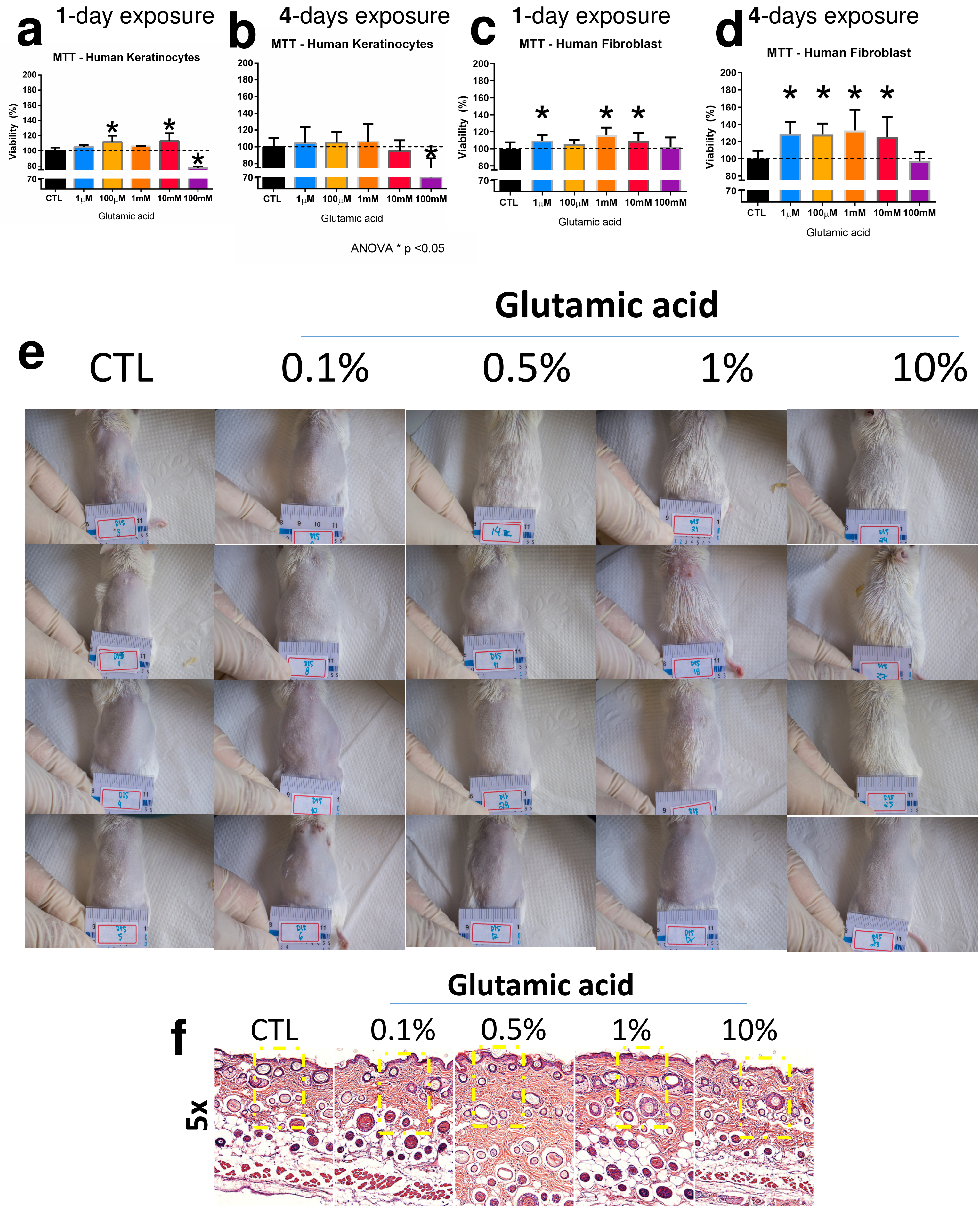

Supplementary Figure 2

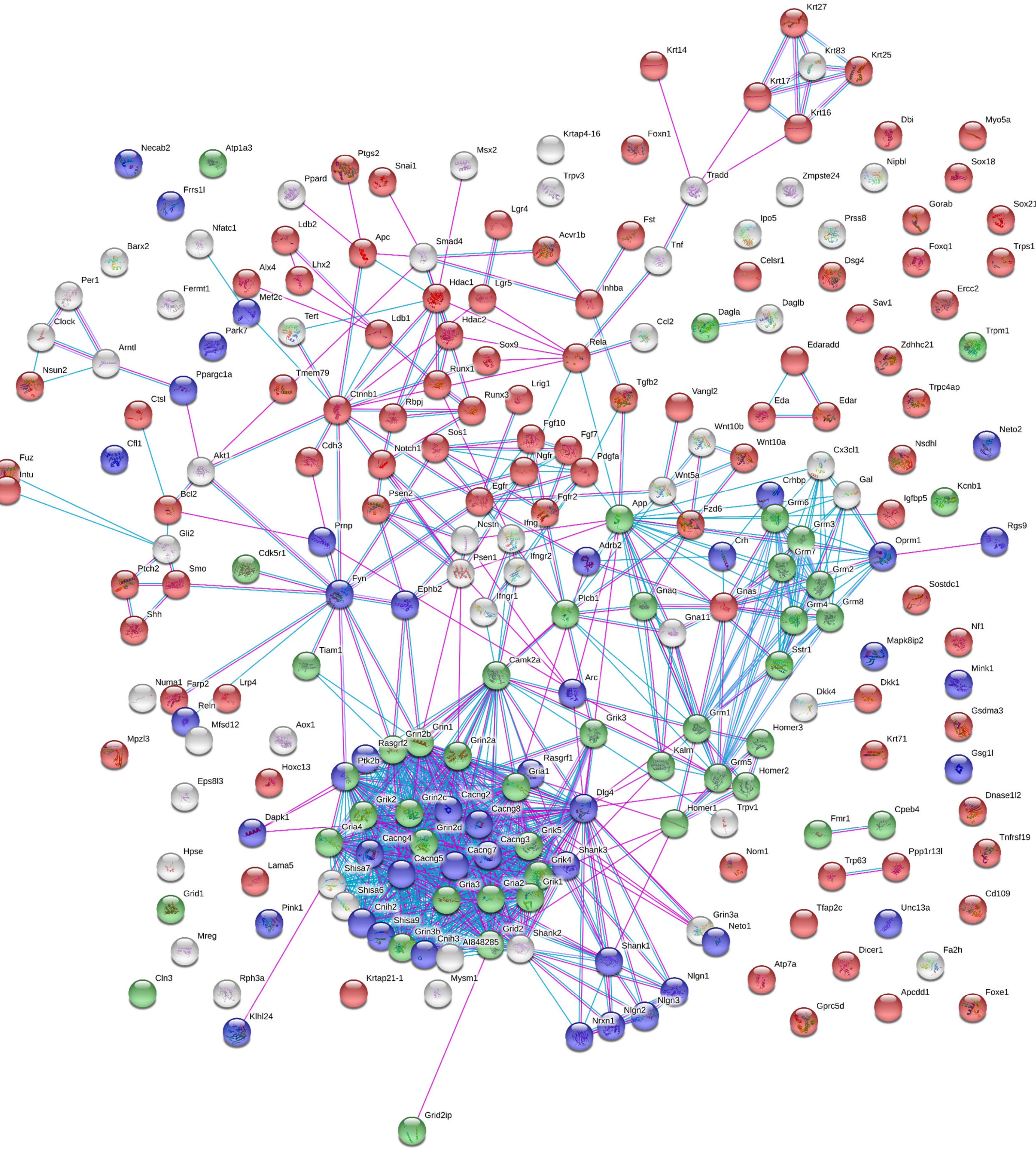

Supplementary Figure 3

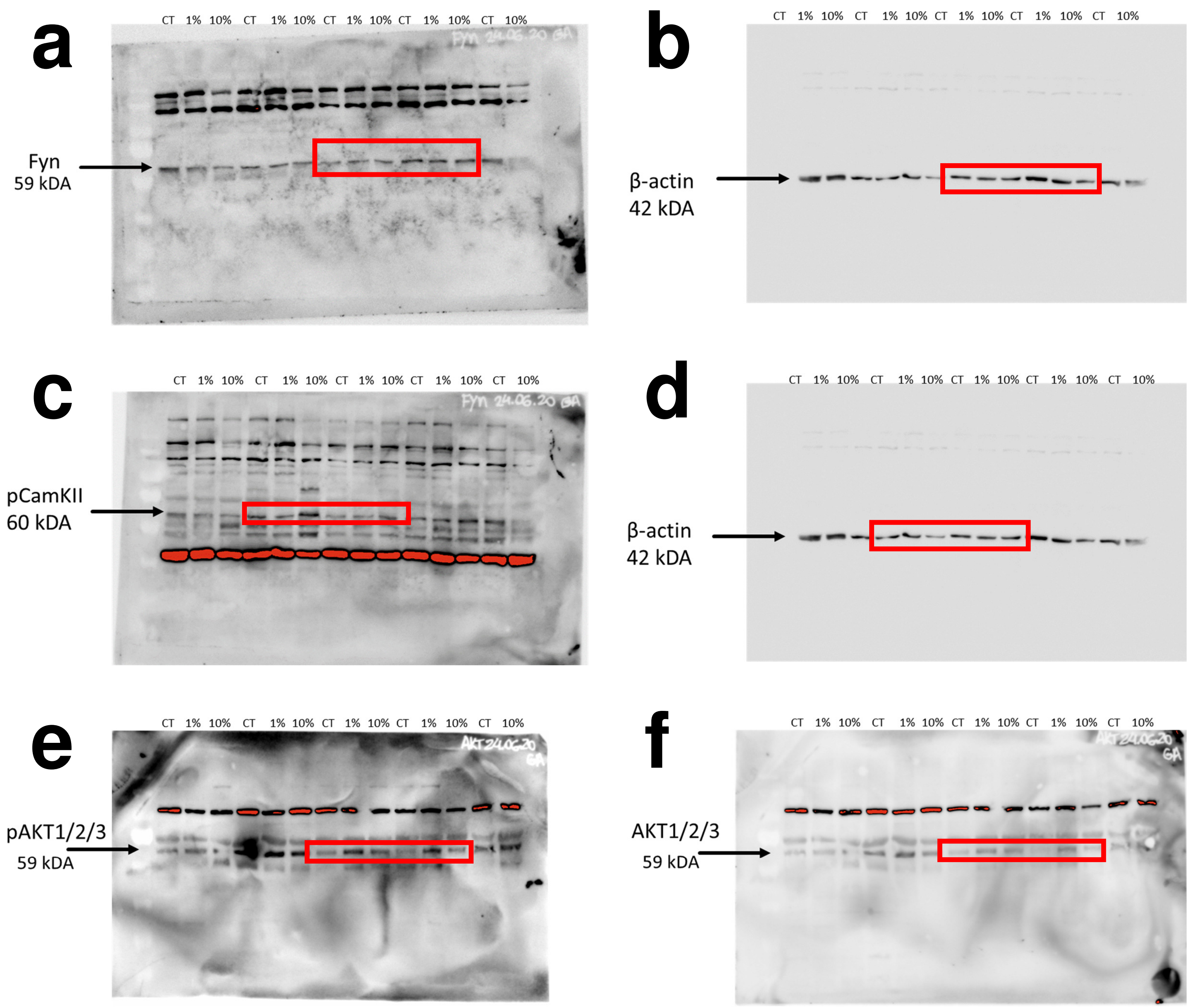

Supplement: Supplementary file 1 — Supplementary Information. [file 41598_2021_94816_MOESM1_ESM.pdf]
